# Protein complex detection based on flower pollination mechanism in multi-relation reconstructed dynamic protein networks

**DOI:** 10.1186/s12859-019-2649-0

**Published:** 2019-03-29

**Authors:** Xiujuan Lei, Ming Fang, Ling Guo, Fang-Xiang Wu

**Affiliations:** 1 School of Computer Science, Shaanxi Normal University, 710119, Xi’an, China; 2 College of Life Sciences, Shaanxi Normal University, 710119, Xi’an, China; 3 Department of Mechanical Engineering and Division of Biomedical Engineering, University of Saskatchewan, Saskatoon, SK S7N 5A9 Canada

**Keywords:** Protein complex, Dynamic protein-protein interaction (PPI) network, Essential protein, Flower pollination algorithm

## Abstract

**Background:**

Detecting protein complex in protein-protein interaction (PPI) networks plays a significant part in bioinformatics field. It enables us to obtain the better understanding for the structures and characteristics of biological systems.

**Methods:**

In this study, we present a novel algorithm, named Improved Flower Pollination Algorithm (IFPA), to identify protein complexes in multi-relation reconstructed dynamic PPI networks. Specifically, we first introduce a concept called co-essentiality, which considers the protein essentiality to search essential interactions, Then, we devise the multi-relation reconstructed dynamic PPI networks (MRDPNs) and discover the potential cores of protein complexes in MRDPNs. Finally, an IFPA algorithm is put forward based on the flower pollination mechanism to generate protein complexes by simulating the process of pollen find the optimal pollination plants, namely, attach the peripheries to the corresponding cores.

**Results:**

The experimental results on three different datasets (DIP, MIPS and Krogan) show that our IFPA algorithm is more superior to some representative methods in the prediction of protein complexes.

**Conclusions:**

Our proposed IFPA algorithm is powerful in protein complex detection by building multi-relation reconstructed dynamic protein networks and using improved flower pollination algorithm. The experimental results indicate that our IFPA algorithm can obtain better performance than other methods.

## Background

Understanding biological processes is an important task in the living organisms. Proteins are vital components in many biological processes, such as metabolism, signaling, transportation and so on. Biological functions are performed by protein complexes composed of proteins interacted with each other, rather than by individual proteins [1, 2]. Detection of protein complexes made great contribution to our knowledge of the molecular mechanisms in cellular life activities. To the best of our knowledge, a large number of works have been done to identify protein complexes from the PPI networks up to now.

As one of the earliest computational methods to predict protein complexes, the Molecular Complex Detection (MCODE) [3] weighted all vertices by using their local neighborhood density and identified the densely connected areas in PPI networks. ClusterONE [4] was utilized to find overlapping protein complexes in the PPI networks. The Clustering-based on Maximal Cliques (CMC) [5] method weighted the interacting protein pairs to identify protein complex. Recent studies TP-WDPIN [6] and NEOComplex [7] were based on the seed-extension idea to mine protein complexes. WG-Cluster [8] considered the edge weights to detect network modules. Markov Clustering (MCL) [9] discovered relatively dense regions based on the random walks. After that, F-MCL [10] used the firefly algorithm into Markov clustering to optimize the parameters and then recognized protein complexes.

It is well known that Gavin et al. [11] introduced the proteins in complexes consist of two types: core and attachment (periphery), namely, core-attachment structure, core represents the proteins that are densely linked and attachment are those proteins that have a few connections to the core. And then, CORE [12] first identified cores and then added proteins that had interactions with the majority of core proteins in the protein complex as attachment proteins. COACH [13] predicted cores in complexes and involved attachments into the cores to obtain protein complexes. Similarly, DCA [14] also used core-attachment feature to identify protein complexes.

In general, the judgment of interactions between two proteins is implemented by using experimental methods. Unfortunately, these methods are not always dependable [1] and it means that this may contain false positive interactions. There are many previous literatures have revealed the fact that the incorporation of additional biological information can improve the accuracy of protein complex prediction to some extent. For example, Zhang et al. [15] proposed CSO method to predict complexes by combining gene ontology (GO) information with PPI networks. InteHC method [16] integrated different types of data sources to predict protein complexes, including PPI data, GO data, gene expression profiles and AP-MS data. Zhao et al. [17] constructed the weighted protein interaction network by using gene expression information for protein complex identification. Zhou et al. [18] utilized GO to measure semantic similarities as the weights.

Since the flower pollination algorithm (FPA) [19] has shown excellent performance in many applications, such as clustering problem [20] and the identification of essential proteins [21], we explore the application of FPA in detecting protein complexes. In this study, we elicit a concept named co-essentiality, whose basic idea is to use the protein essentiality to find essential edges. Then the multi-relation reconstructed dynamic protein networks are built by combining heterogeneous topology and biology information. Next, those closely linked proteins are grouped together as the cores. Finally, based on the core–periphery structure, the modified FPA algorithm is developed to find the optimal pollination plants for pollen, which means that the peripheries attach to the best core to form the predicted protein complex. The experiments between IFPA and several typical algorithms including MCODE, MCL, ClusterONE, CSO, COACH and CORE are performed on three different PPI networks, and the results demonstrate that IFPA algorithm is more robust and powerful than those existing methods in protein complexes recognition.

The remaining part of this paper is organized as follows. Section 2 (Methods) elucidates our proposed new algorithm called IFPA. Section 3 (Results and Discussion) provides the exhaustive analysis and descriptions for the experiments. Finally, Section 4 (Conclusions) is the summary of this study.

## Methods

The PPI network can be represented generally as an undirected graph, and the proteins are treated as nodes and the interactions are considered as edges. Here, the static PPI networks are converted into the multi-relation reconstructed dynamic PPI networks. And then we apply IFPA to add attachments to the appropriate cores based on the core-attachment structure.

### Building multi-relation reconstructed dynamic PPI networks

The availability of gene expression data enables researchers to reveal the dynamics of molecular networks and improve the identification of protein complexes [22–24]. Hence, based on the study [25], the time course gene expression data is integrated into original static PPI networks to generate dynamic PPI subnetworks so that we can capture the dynamics of protein complexes, that is to say, we split the original static PPI network (OSPN) into twelve dynamic PPI subnetworks (DPSNs), in which all interactions in a DPSN can occur simultaneously, and then perform complex discovery on each DPSN.

First, we use three-sigma method [25] in order to construct dynamic PPI subnetworks with time series gene expression data. The gene expression data involves three metabolic cycles and each cycle contains twelve timestamps. A protein *v* is considered to be active in DPSN if its gene expression value is not less than the active threshold *Active_Th*(*v*):

1 $$ Active\_ Th(v)=\mu (v)+\kern0.5em 3\sigma (v)\left(1-F(v)\right) $$

2 $$ F(v)=\kern0.5em \frac{1}{1+\kern0.5em {\sigma}^2(v)} $$

where *μ*(*v*) is the algorithmic mean of gene expression values of *v* over times 1 to *n* and *σ*(*v*) is the standard deviation of its gene expression values. For each protein, three-sigma method is used to calculate the active threshold *Active_Th*(*v*). A original PPI network can be described as an undirected graph *G*(*V*, *E*), where *V* denotes node set that are proteins and *E* presents edge set that are their connections. And the dynamic PPI network can be represented as *G*_*t*_ (*V*_*t*_, *E*_*t*_) at timestamp *t* (*t* = 1,2, …, *n*). At a certain time point, if two proteins *v*_*i*_ and *v*_*j*_ are active and interact with each other in the original static PPI network, then there is a connection between protein *v*_*i*_ and *v*_*j*_ in a DPSN. After that, twelve dynamic PPI subnetworks are constructed from the original static PPI network.

**Fig. 1:**
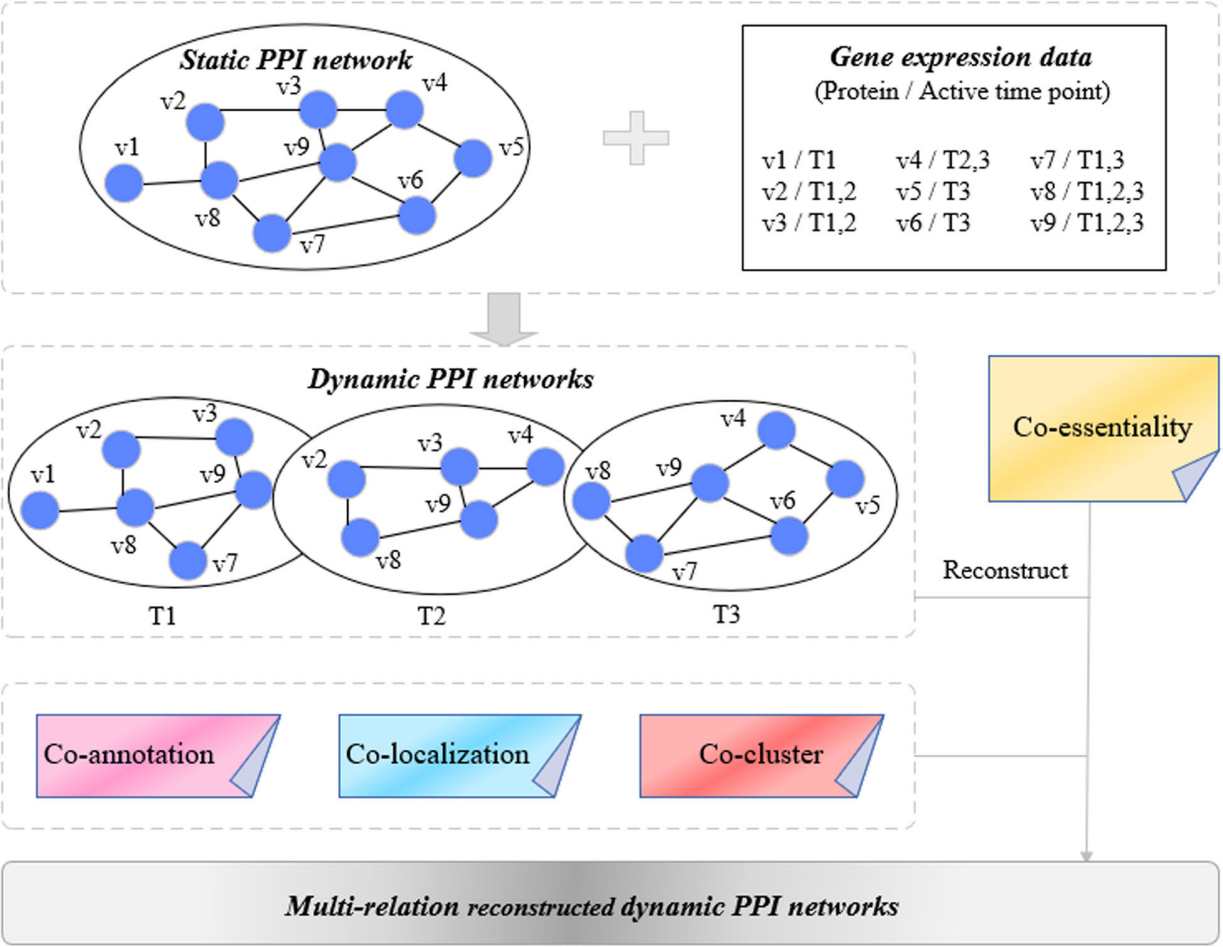
An example of multi-relation reconstructed dynamic PPI networks construction

Moreover, integrating heterogeneous data source into a single network can enhance the reliability of networks, which inspires us that assigning the suitable weights to edges can strengthen the confidence of interactions, and the implementation will be discussed in the following. Figure 1 illustrates an example of multi-relation reconstructed dynamic PPI networks construction.

- **Definition 1** (Co-essentiality) Essential proteins are indispensable for the survival of an organism. Then we can believe that the interaction between two essential proteins is also necessary. Hence, a concept based on essential protein is extended to measure the essentiality between two proteins, and the essentiality values are considered as their weights.

Before giving the concept of co-essentiality, we first elaborate the definition of an essential edge. Given two proteins *v*_*i*_ and *v*_*j*_, the edge between them is considered as an essential edge if both of *v*_*i*_ and *v*_*j*_ are the essential proteins, similarly, the edge between them is considered as an uncertain edge if *v*_*i*_ or *v*_*j*_ is the essential protein, and the edge between them is considered as a nonessential edge if neither of *v*_*i*_ and *v*_*j*_ is the essential protein. Only the essential edges are taken into account to reconstruct the networks here. And *ee*_*ij*_ is the essential edge between *v*_*i*_ and *v*_*j*_, the *co-essentiality* between these two proteins can be represented as follows.

3 $$ co- essentiality\kern0.5em \left(i,j\right)=\kern0.5em \frac{ESS_{ij}}{sum\kern0.5em \left({ESS}_j\right)} $$

where *ESS*_*ij*_ denotes the weight value of essential edge which equals to one and sum(*ESS*_*j*_) denotes the sum of the weight values of a column.

- **Definition 2** (Co-localization) Given two interacting proteins *v*_*i*_ and *v*_*j*_, the interaction between them will be more reliable if *v*_*i*_ and *v*_*j*_ exist in same subcellular location, its *co-localization* is defined by the following equation.

4 $$ co- localization\kern0.5em \left(i,j\right)=\kern0.5em \frac{{\left|{SCL}_i\kern0.5em \cap \kern0.5em {SCL}_j\right|}^2}{\mid {SCL}_i\mid \cdot \mid {SCL}_j\mid } $$

where |*SCL*_*i*_| and |*SCL*_*j*_| are the number of subcellular location of proteins *v*_*i*_ and *v*_*j*_, respectively.

- **Definition 3** (Co-annotation) Given two interacting proteins *v*_*i*_ and *v*_*j*_, they have the similar function if there are some common GO annotations between *v*_*i*_ and *v*_*j*_, its *co-annotation* is calculated as follows.

5 $$ co- annotation\left(i,j\right)=\frac{{\left|{GO}_i\cap {GO}_j\right|}^2}{\left|{GO}_i\right|\cdot \left|{GO}_j\right|} $$

where |*GO*_*i*_| and |*GO*_*j*_| are the number of GO annotations of proteins *v*_*i*_ and *v*_*j*_, respectively.

- **Definition 4** (Co-cluster) Given two interacting proteins *v*_*i*_ and *v*_*j*_, its *co-cluster* is measured by using the edge clustering coefficient (ECC) [26] as follows.

6 $$ co- cluster\left(i,j\right)=\frac{Z_{ij}}{\mathit{\min}\left\{\left|{N}_i\right|-1,\left|{N}_j\right|-1\right\}} $$

where *Z*_*ij*_ represents the number of triangles built on edge (*v*_*i*_, *v*_*j*_), |*N*_*i*_| and |*N*_*j*_| are the degrees of protein *v*_*i*_ and *v*_*j*_, respectively.

Multiple relation defined above are used to weight the networks. The multi-relation value between *v*_*i*_ and *v*_*j*_ is stands for as follows.

$$ multi- relation\left(i,j\right)= co- essentiality\left(i,j\right)+ co- localization\left(i,j\right) $$

7 $$ + co- annotation\left(i,j\right)+ co- cluster\left(i,j\right) $$

These multi-relation values are regarded as the weights of edges *W*(*i*,*j*) to upgrade the credibility of interactions. For an edge, its normalized *W*(*i*,*j*) value *NW*(*i*,*j*) is expressed by the following formula.

8 $$ NW\left(i,j\right)=\frac{multi- relation\left(i,j\right)}{num\left( multi- relation\right)} $$

where *num(multi-relation)* is the total number of the network relations, i.e., the four kinds of relations including coessentiality, colocalization, coannotation, cocluster and the networks are reconstructed by mixing them. Eventually, the dynamic PPI subnetworks (DPSNs) are switched into the multi-relation reconstructed dynamic PPI networks (MRDPNs).

### Finding cores

As we all know that protein complex core should be a densely connected subgraph in the PPI network. Thus, we pick the seed proteins in the first stage, and extend seed proteins to the cores in the second stage.

- **Definition 5** (Weighted Degree) The proteins with weighted degree greater than average weighted degree are sorted in descending order as the candidate core set *CC*. The weighted degree of a protein *i* in the MRDPN is the number of interactions in which this protein is involved, which can be expressed as follows.

9 $$ Weighted\ Degree(i)=\sum \limits_j interactions\left(i,j\right) $$

Let first node in the candidate core set *CC* be a seed protein which plays an irreplaceable role in protein complex. The neighbors of the seed protein are inserted into a core set when the condition that the density of core set is greater than a given threshold *DT* is satisfied. The threshold *DT* will be discussed in the next section.

- Definition 6 (Density) The density of core set *CS* can measure how close the core is, and its definition is as follows.

10 $$ Density(CS)=\frac{2\times {\sum}_{\left(i,j\right)} NW\left(i,j\right)}{\left| CS\right|\cdot \left(\left| CS\right|-1\right)} $$

where |*CS*| denotes the number of nodes in core set. Initially, core set *CS* contains one seed protein *i*. A neighbor of seed protein is added to the core set if adding it can make the *Density*(*CS*) greater than the threshold *DT*. This process is repeated until all neighbors of seed protein are sought and the predicted core is generated. Once a complex core is completed, all nodes in it will be labeled with “1” and cannot be extended into any other complex cores. This process will stop when the *CC* is empty.

### Finding peripheries

Since the core plays a central role, the periphery plays a supporting role. The key idea behind our presented IFPA algorithm is to utilize the pollination mechanism to mimic the process of pollen falling on suitable flowers, which is completely different from other general methods. In this subsection, we first give a brief introduction to the flower pollination algorithm (FPA) [19], and then we find the optimal cores for peripheries by ameliorating it.

FPA is a nature-inspired optimization algorithm that comprises two main patterns, that is global pollination and local pollination. The global pollination can be represented as:

$$ {x}_i^{t+1}={x}_i^t+L\left({x}_i^t-G\right)\ (11) $$

where ${x}_i^t$ is the pollen *i* at iteration *t*, and *G* is the current best solution. The parameter *L* is the strength of the pollination, namely a step size, we use a Lévy flight to represent that insects move over a long distance with various distance steps. That is, *L* is greater than 0 and obeys the Lévy distribution:

$$ L\sim \frac{\lambda \varGamma \left(\lambda \right)\sin \left(\pi \lambda /2\right)}{\pi}\frac{1}{s^{1+\lambda }},\left(s\gg {s}_0>0\right)\ (12) $$

where *Γ*(*λ*) is the standard gamma function. The local pollination can be defined as:

$$ {x}_i^{t+1}={x}_i^t+\varPsi \left({x}_j^t-{x}_k^t\right)\ (13) $$

where ${x}_j^t$ and ${x}_k^t$ are pollen from the different flowers of the same plant species. This substantially simulates the flower constancy in a limited neighborhood. Mathematically, if ${x}_j^t$ and ${x}_k^t$ come from the same plant species or select from the same population, this can be seen as a local random walk if *Ψ* obeys the uniform distribution of 0 to 1.

**Fig. 2:**
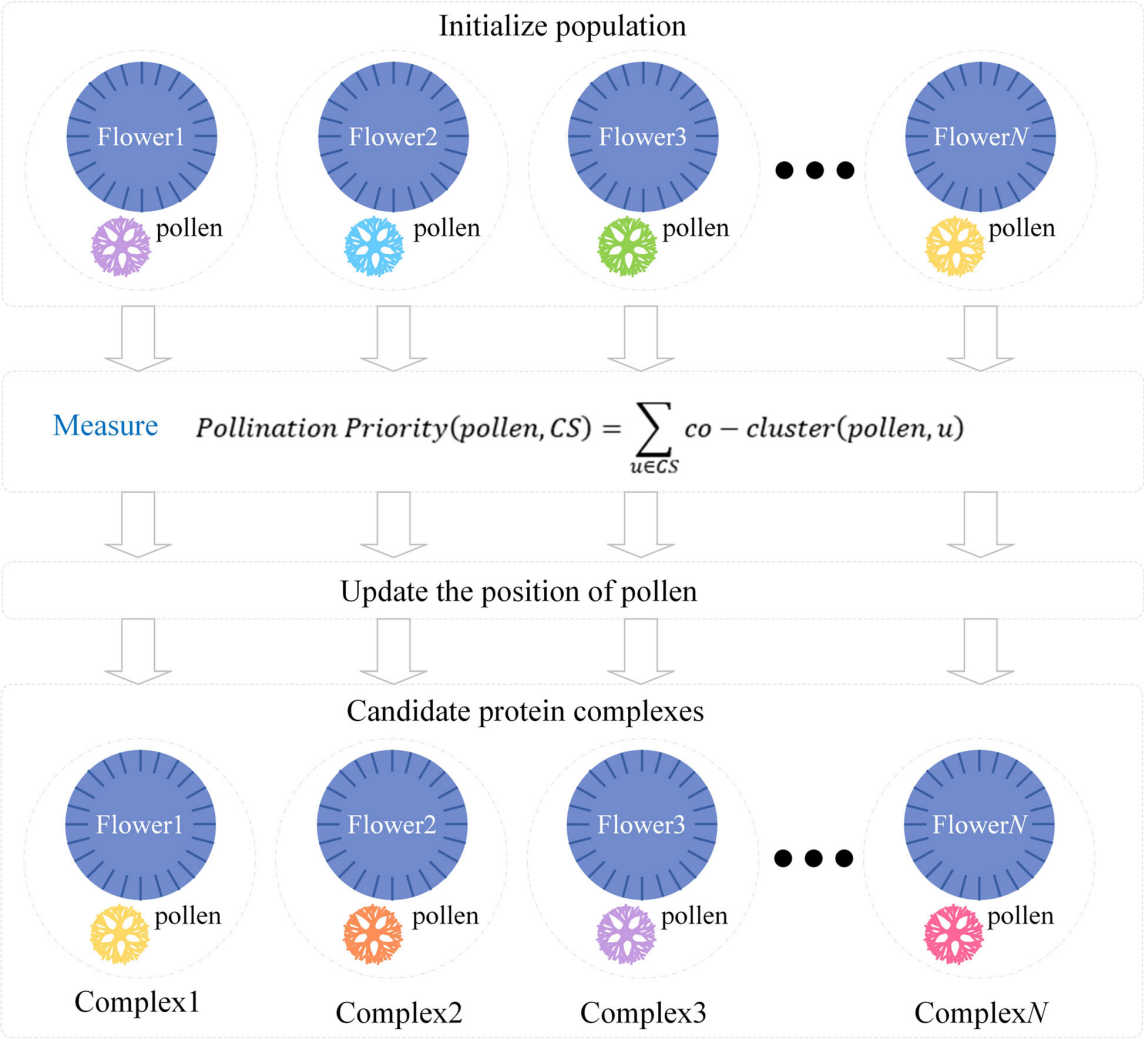
The overall framework of our presented IFPA algorithm

Then, we use IFPA which is an advanced version of FPA algorithm to find the closest cores for peripheries, which is equivalent to finding the most satisfactory flowering plants for pollen. The workflow of IFPA algorithm is shown in Fig. 2. Those proteins not included in the core set are considered as the candidate pollen. In IFPA algorithm, the pollen corresponds to attachments and the pollination plants correspond to cores. The pollen position equals the core sequence numbers. The update of pollen position is expressed as follows.

$$ {S}_{i,j}^{t+1}=\left\{\begin{array}{c}\ {S}_{i,j}^t, if\ {Pollination\ Priority}_{i,j}> Thr\ \\ {}\  randperm\left( Num,d\right), otherwise\end{array}\right.\ (14) $$

where *Thr* denotes a threshold and it is set as 0.2 here. The function of *randperm* is to return an integer from one to *Num* which means to find new core sequence number, and *Num* is the number of cores and the value of *d* is one.

**Definition 7** (Pollination Priority) As a part of an entire protein complex, the attachments maintain relatively close relationship with the core, we call this relationship as *pollination priority*. The “*pollination priority*” of a pollen to its core set *CS* is represented as follows.

$$ Pollination\ Priority\left( pollen, CS\right)=\sum \limits_{u\in CS} co- cluster\left( pollen,u\right)\ (15) $$

where *u* is the protein in core set *CS*. The pollination priority depends on the affinity between the pollen and the flowers. The closer the relationship between pollen and a flower, the higher priority it pollinates on this flower. In the update procedure, if the pollen can find a flower that makes the value of *pollination priority* better, then the pollen falls on this flower, otherwise, the pollen finds a new flower to pollinate.

Finally, we further merge all the candidate protein complexes mined in twelve subnetworks and filter highly overlapping complexes, as our final predicted protein complexes. Algorithm 1 outlines the implementation process of our IFPA method.

## Results and discussion

### Datasets

Three popular datasets, i.e., DIP [27], MIPS [28] and Krogan [29], are used to verify our proposed IFPA algorithm. The DIP dataset contains 5028 proteins and 22,302 interactions, the MIPS dataset composes of 4546 proteins and 12,319 interactions, and the Krogan dataset includes 2674 proteins and 7075 interactions. The gene expression data is obtained from GEO [30] and the dataset contains 9336 genes in three cell life cycles, each cycle having twelve time points. The dynamic PPI networks are built by combining original static PPI networks with gene expression data and the details of dynamic PPI networks on three datasets are presented in Table 1.

**Table 1:** The number of proteins and interactions in dynamic PPI networks on three datasets

Dataset	Timestamp *t*											
	1	2	3	4	5	6	7	8	9	10	11	12
DIP												
Protein	860	1029	863	671	645	598	530	1000	1194	638	690	489
Interaction	1103	1608	1337	839	835	752	627	1861	2447	950	1026	569
MIPS												
Protein	737	897	781	583	570	531	470	839	1014	523	616	402
Interaction	1097	1443	1183	754	684	642	504	1238	1637	878	1207	700
Krogan												
Protein	336	379	320	256	206	189	202	580	626	304	330	250
Interaction	334	464	331	234	210	184	213	1025	1081	314	373	258

The protein subcellular localization dataset is downloaded from the COMPARTMENTS database [31]. There are eleven subcellular localizations as follows: Cytoskeleton, Golgi apparatus, Peroxisome, Nucleus, Extracellular space, Vacuole, Cytosol, Endosome, Mitochondrion, Plasma membrane, Endoplasmic reticulum. After preprocessing, it still includes 6892 subcellular localization records. The GO information was gained from the SGD database [32]. There are 1285 essential proteins are collected from the following databases: MIPS [33], SGD [32], DEG [34], and SGDP (http://sequence.stanford.edu/group/yeast_deletion_project). CYC2008 [35] is used as the benchmark dataset which contains 408 protein complexes.

### Evaluation metrics

The most commonly used evaluation metrics are used in our experiments and their specific definitions are described below.

**Definition 8** (Overlapping Score) Given a predicted protein complex *P* and a known protein complex *K*, the Overlapping Score (*OS*) between *P* and *K* is defined as follows.

$$ OverlappingScore\left(P,K\right)=\frac{{\left|{V}_P\cap {V}_K\right|}^2}{\mid {V}_P\mid \cdot \mid {V}_K\mid }(16) $$

where |*V*_*P*_ ∩ *V*_*K*_| is the number of common proteins in the predicted protein complex *P* and the known protein complex *K*, |*V*_*P*_| is the size of the predicted complex and |*V*_*K*_| is the size of the known complex. If *OS* ≥ 0.2, we consider that the predicted complex matches with the real one.

**Definition 9** (Sensitivity, Specificity and F-measure) Sensitivity (*Sn*), Specificity (*Sp*) and *F-measure* are represented as follows.

$$ Sn=\frac{TP}{TP+ FN}\ (17) $$

$$ Sp=\frac{TP}{TP+ FP}\ (18) $$

$$ F- measure=\frac{2\cdot Sn\cdot Sp}{Sn+ Sp}(19) $$

where *TP* is the number of the predicted complexes which are matched with the known complexes, *FN* is the number of known complexes which are not matched with any predicted complexes, and *FP* is the number of the predicted complexes which are not matched with any known complexes. *F-measure* is a comprehensive metric combined sensitivity and specificity.

**Definition 10** (*p-value*) In order to estimate the statistical significance of detected protein complexes, the researchers annotate their biological functions by using *p-value*. Given a predicted protein complex, the *p-value* [36] is the probability that a protein complex is enriched by a given functional group by chance. Let *k* is the number of proteins of the functional group in the complex, *N* is the size of the whole PPI network, *C* is the size of a protein complex and *F* is the size of a functional group in the network. And the *p-value* is defined as follows.

$$ p- value=1-\sum \limits_{i=0}^{k-1}\frac{\left(\genfrac{}{}{0pt}{}{F}{i}\right)\left(\genfrac{}{}{0pt}{}{N-F}{C-i}\right)}{\left(\genfrac{}{}{0pt}{}{N}{C}\right)}\ (20) $$

The *p-value* is used to evaluate the biological relevance of the predicted protein complexes. Generally, the protein complex is considered to be meaningless if its *p-value* is greater than 0.01. And for a complex, the smaller its *p-value* is, the more biological significance it has.

### Parameter analysis

**Fig. 3:**
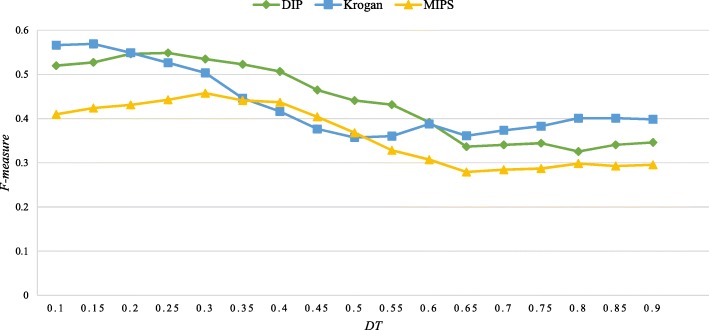
The impact of *DT* on the performance of IFPA in terms of *F-measure*

The density threshold *DT* decides whether a protein could be merged into the current core. How to choose a relatively suitable *DT* should be carefully considered to achieve better performance of our IFPA algorithm. Thus, varying *DT* from 0.1 to 0.9 with the interval 0.05, we calculate *F-measure* to observe the effect of the variation of *DT* on the performance of our IFPA algorithm, so as to choose the relatively appropriate *DT*, as shown in Fig. 3. Obviously, all three datasets show similar trends in most cases from Fig. 3. Especially, the major evaluation metrics *F-measure* obtains the better value when *DT* is set as 0.25 based on the both DIP and MIPS datasets and when *DT* is set as 0.15 based on the Krogan dataset, which means that IFPA is more effective. For this reason, the density threshold *DT* is set as 0.25 in DIP and MIPS datasets and 0.15 in Krogan dataset.

### Performance comparison

**Fig. 4:**
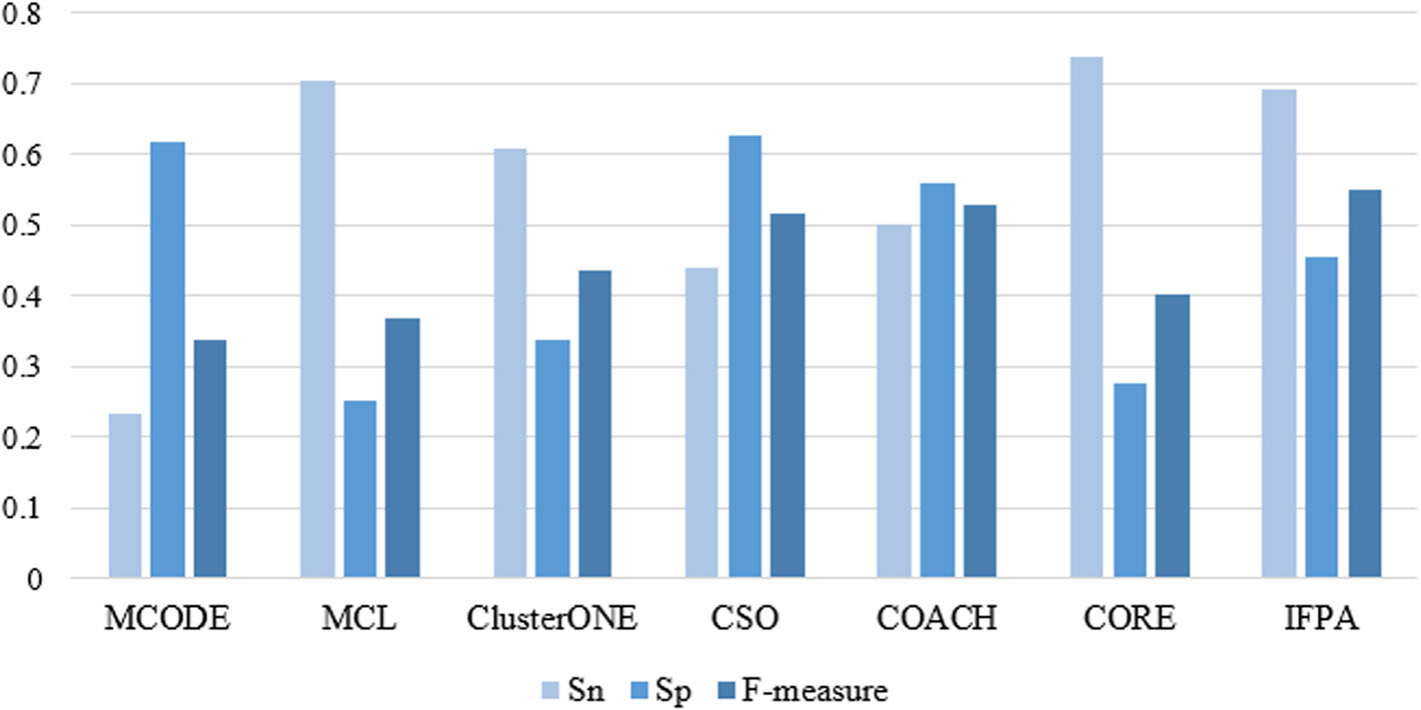
Comparative performance of IFPA and other methods on DIP dataset

**Fig. 5:**
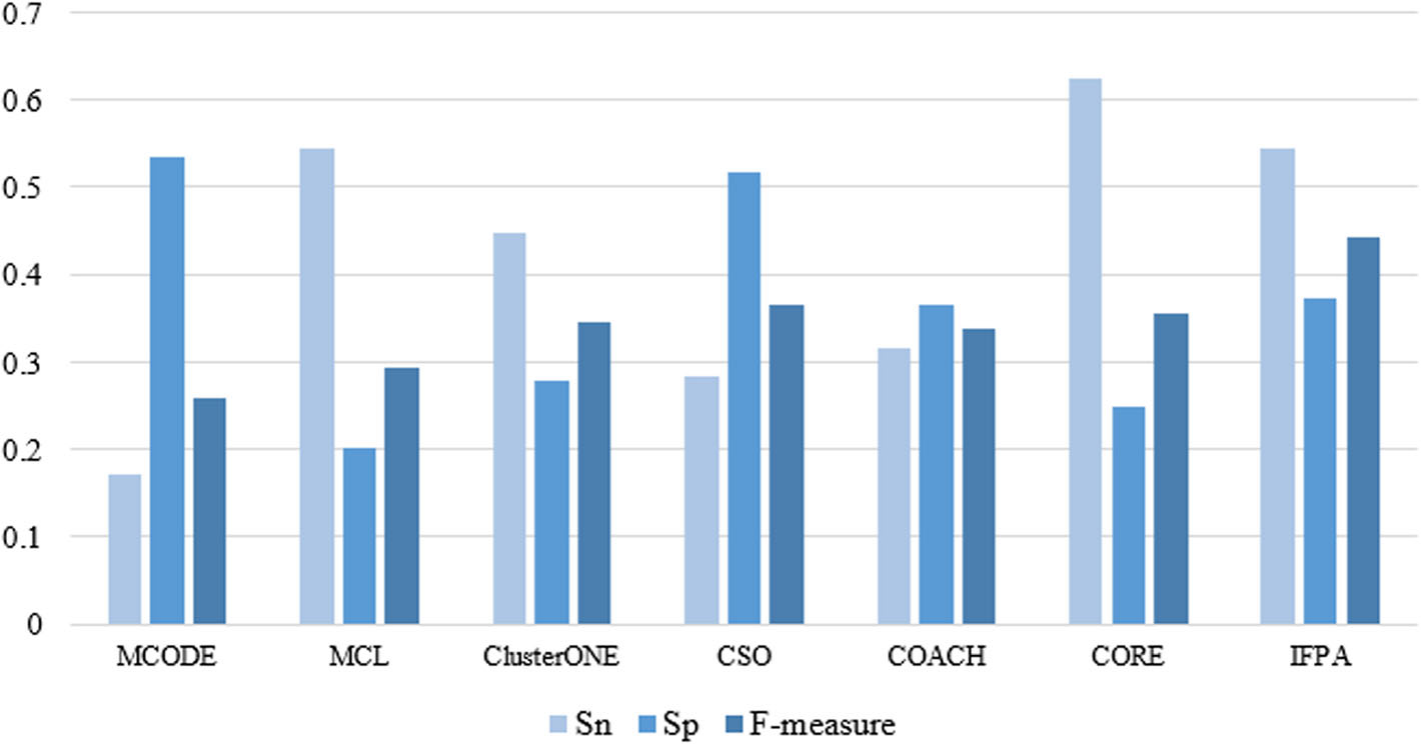
Comparative performance of IFPA and other methods on MIPS dataset

**Fig. 6:**
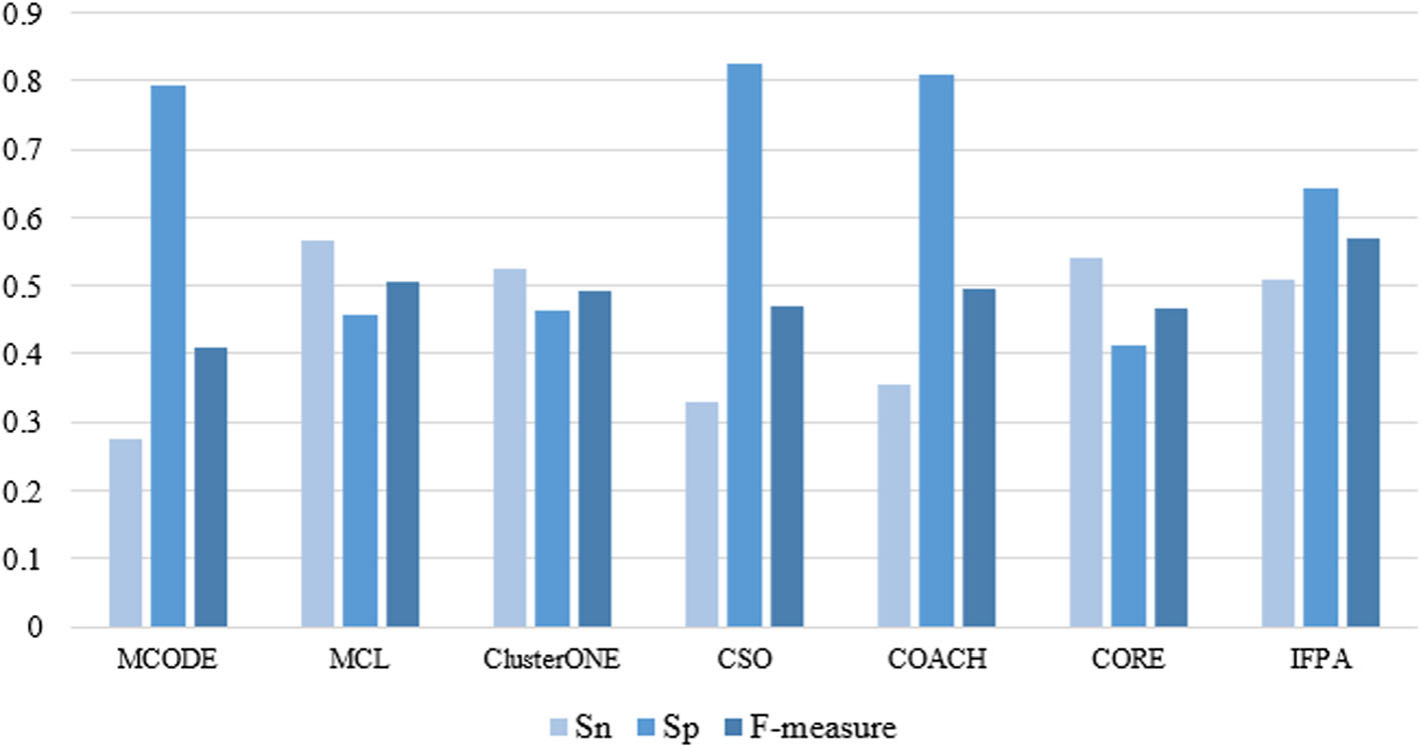
Comparative performance of IFPA and other methods on Krogan dataset

To prove the validity of our proposed algorithm, we compare IFPA algorithm with MCODE, MCL, ClusterONE, CSO, COACH and CORE on three different PPI networks. Figures 4, 5, 6 show the overall comparison in terms of *Sn*, *Sp* and *F-measure* based on DIP, MIPS and Krogan datasets, respectively. Apparently, IFPA algorithm yields the best *F-measure* in comparison with other existing methods in all datasets, which means that our IFPA method remarkably outperforms other methods. Besides, in Table 2, *PC* denotes the total number of detected protein complexes, *MPC* is the number of detected protein complexes which were matched, *MKC* represents the number of matched known protein complexes, *Perfect* denotes *OS* = 1 which means that the predicted protein complexes are perfectly matched with the known protein complexes. As shown in Table 2, the protein complexes detected by our IFPA method dominates other methods in the aspects of *Perfect* both in DIP and MIPS datasets.

**Table 2:** Comparative performance of IFPA and other methods on three datasets

Dataset	Algorithm	PC	MPC	MKC	Perfect
DIP	MCODE	165	102	70	6
	MCL	1541	386	245	14
	ClusterONE	972	329	197	15
	CORE	1517	420	259	39
	CSO	342	214	136	11
	COACH	474	265	144	13
	IFPA	935	425	219	47
MIPS	MCODE	135	72	60	4
	MCL	1259	254	196	17
	ClusterONE	744	208	152	17
	CORE	1217	303	225	29
	CSO	246	127	87	6
	COACH	396	145	92	5
	IFPA	772	288	167	32
Krogan	MCODE	160	127	73	10
	MCL	658	300	178	40
	ClusterONE	585	271	161	28
	CORE	677	279	172	39
	CSO	189	156	89	10
	COACH	221	179	85	11
	IFPA	447	288	131	21

Many of our detected protein complexes have a good match with the known protein complexes. We consider a detected complex to be biologically significant if its *p-value* is less than 0.01. In order to confirm the biological significance of detected protein complexes, the *p-value* is calculated by using the tool GO::TermFinder (https://www.yeastgenome.org/cgi-bin/GO/goTermFinder.pl). We randomly select some predicted protein complexes to calculate their *p-value* concerning Biological Process ontologies based on Krogan dataset, as shown in Table 3. From Table 3, all of these detected protein complexes obtain smaller *p-value* and it demonstrates that the protein complexes predicted by our IFPA method have strong biological significance. The predicted complexes with strong biological significance can provide help for biology researches to some extent.

**Table 3:** Function enrichment analysis of predicted protein complexes detected on Krogan dataset

No.	Gene Ontology term	*p-value*	Cluster frequency	Genes annotated to the term
1	intra-Golgi vesicle-mediated transport	1.91e-16	100.0%	COG3/YER157W, COG7/YGL005C, COG1/YGL223C, COG2/YGR120C, COG8/YML071C, COG6/YNL041C, COG4/YPR105C
2	polyadenylation-dependent snoRNA 3’-end processing	3.32e-18	100.0%	RRP43/YCR035C, RRP45/YDR280W, MTR3/YGR158C, SKI6/YGR195W, LRP1/YHR081W, RRP40/YOL142W, RRP6/YOR001W
3	exonucleolytic trimming involved in rRNA processing	5.02e-20	100.0%	RRP43/YCR035C, RRP42/YDL111C, RRP45/YDR280W, MTR3/YGR158C, SKI6/YGR195W, RRP4/YHR069C, LRP1/YHR081W, CSL4/YNL232W
4	negative regulation of gluconeogenesis	3.00e-17	100.0%	GID7/YCL039W, RMD5/YDR255C, VID30/YGL227W, VID28/YIL017C, FYV10/YIL097W, GID8/YMR135C
5	chromatin disassembly	3.83e-20	100.0%	HTL1/YCR020W-B, RSC6/YCR052W, RTT102/YGR275W, STH1/YIL126W, RSC58/YLR033W, SFH1/YLR321C, RSC2/YLR357W, NPL6/YMR091C
6	positive regulation of transcription from RNA polymerase I promoter	5.96e-16	87.5%	UTP4/YDR324C, UTP5/YDR398W, UTP8/YGR128C, UTP9/YHR196W, UTP10/YJL109C, UTP15/YMR093W, NAN1/YPL126W

**Fig. 7:**
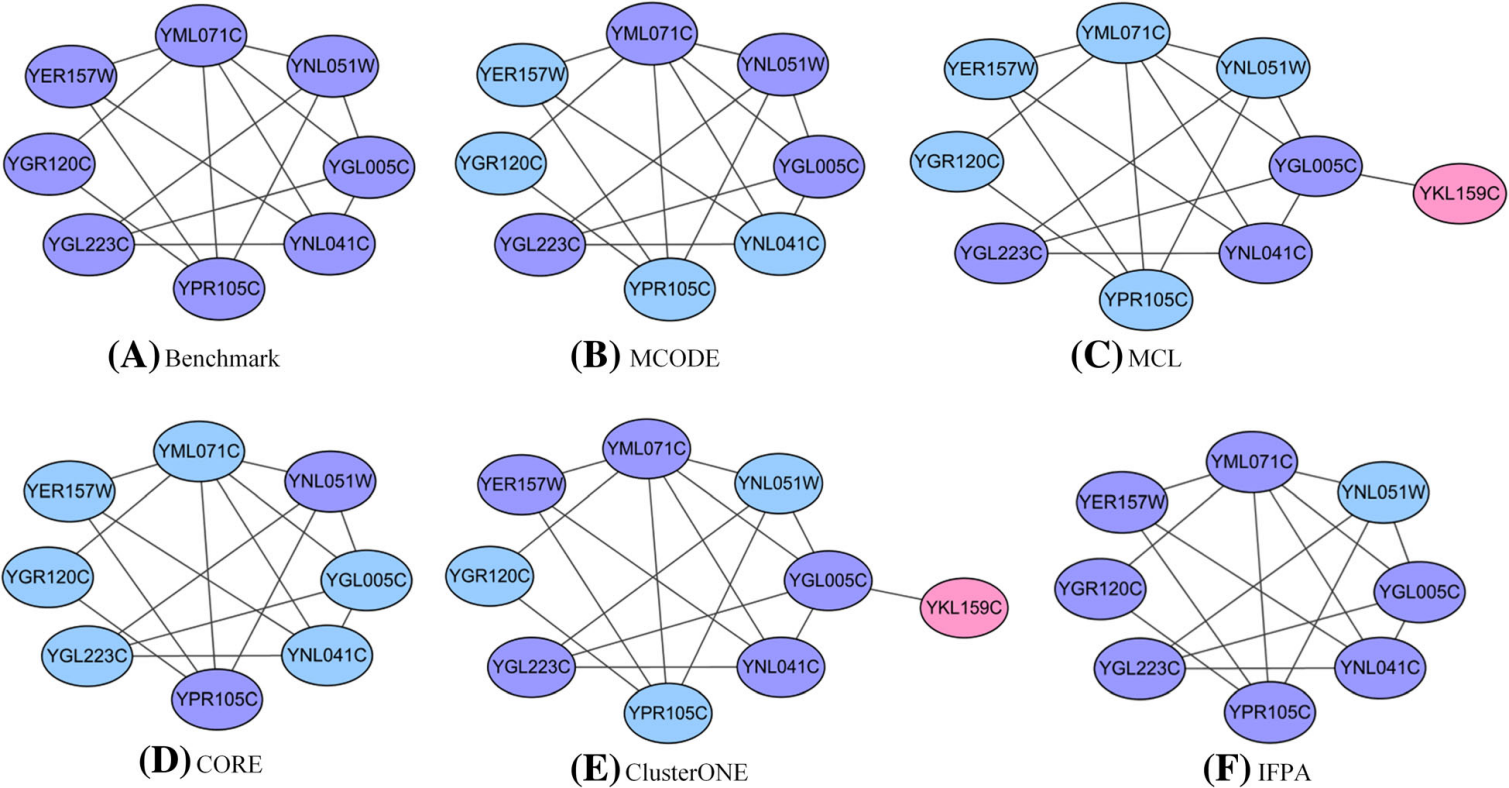
The golgi transport complex detected by different methods

Figure 7 visualizes an example of predicted protein complex named golgi transport complex in Krogan dataset so as to display the detection result more obviously. Figure 7 (A) displays a benchmark protein complex, Fig. 7 (B), (C), (D), (E) and (F) illustrate the identified protein complexes by MCODE, MCL, CORE, ClusterONE and IFPA, respectively. The purple nodes are the correctly identified proteins, the blue nodes are proteins that are not recognized, and the pink nodes are the wrongly identified proteins. From Fig. 7, we can see that MCODE and CORE correctly identifies four and two proteins, respectively. And MCL identifies a total of four proteins including one that is misidentified. Albeit ClusterONE recognizes more proteins, it also has misidentifications. IFPA successfully detects the most proteins and all of them are correct, indicating that our predicted complex match very well with benchmark complex and our IFPA method is more accurate than other comparative methods.

## Conclusions

Identification of protein complexes from PPI networks is distinctly important in proteomics. In this study, a flower pollination mechanism-based method is proposed to detect protein complexes in multi-relation reconstructed dynamic protein networks. To begin with, we build multi-relation reconstructed dynamic protein networks. Then, according to the core–periphery structure, we group the closely connected proteins as the cores and apply IFPA algorithm to attach peripheries to the optimal cores to form the predicted protein complexes. IFPA algorithm has been carried out on three different multi-relation reconstructed dynamic PPI networks and the experimental results demonstrate that our IFPA algorithm can obtain better clustering performance compared with other methods in most cases. The protein complexes we detected are likely to help the biologists gain some useful biological insights.

## Abbreviations

ECC: Edge clustering coefficient
FPA: Flower pollination algorithm
GO: Gene ontology
OS: Overlapping Score
PPI: Protein-protein interaction

## References

[1] von Mering C, Krause R, Snel B, Cornell M, Oliver SG, Fields S, Bork P (2002). Comparative assessment of large-scale data sets of protein-protein interactions. Nature.

[2] De Las Rivas J, Fontanillo C (2010). Protein-protein interactions essentials: key concepts to building and analyzing interactome networks. PLoS Comput Biol.

[3] Bader GD, Hogue CWV (2003). An automated method for finding molecular complexes in large protein interaction networks. BMC bioinformatics.

[4] Nepusz T, Yu H, Paccanaro A (2012). Detecting overlapping protein complexes in protein-protein interaction networks. Nat Methods.

[5] Liu G, Wong L, Chua HN (2009). Complex discovery from weighted PPI networks. Bioinformatics.

[6] Lei X, Zhang Y, Cheng S, Wu F-X, Pedrycz W (2018). Topology potential based seed-growth method to identify protein complexes on dynamic PPI data. Inf Sci.

[7] Ma C-Y, Y-PP C, Berger B, Liao C-S (2017). Identification of protein complexes by integrating multiple alignment of protein interaction networks. Bioinformatics.

[8] Lecca P, Re A (2015). Detecting modules in biological networks by edge weight clustering and entropy significance. Front Genet.

[9] Van Dongen S. Graph clustering by flow simulation: Phd thesis University of Utrecht; 2000.

[10] Lei X, Wang F, Wu F-X, Zhang A, Pedrycz W (2016). Protein complex identification through Markov clustering with firefly algorithm on dynamic protein-protein interaction networks. Inf Sci.

[11] Gavin A-C, Aloy P, Grandi P, Krause R, Boesche M, Marzioch M, Rau C, Jensen LJ, Bastuck S, Dumpelfeld B (2006). Proteome survey reveals modularity of the yeast cell machinery. Nature.

[12] Leung HCM, Xiang Q, Yiu SM, Chin FYL (2009). Predicting protein complexes from PPI data: a core-attachment approach. J Comput Biol.

[13] Wu M, Li X, Kwoh C-K, Ng S-K (2009). A core-attachment based method to detect protein complexes in PPI networks. BMC Bioinformatics..

[14] Shen X, Yi L, Jiang X, He T, Yang J, Xie W, Hu P, Hu X (2017). Identifying protein complex by integrating characteristic of core-attachment into dynamic PPI network. PLoS One.

[15] Zhang Y, Lin H, Yang Z, Wang J, Li Y, Xu B (2013). Protein complex prediction in large ontology attributed protein-protein interaction networks. IEEE/ACM Transactions on Computational Biology and Bioinformatics..

[16] Wu M, Xie Z, Li X, Kwoh C-K, Zheng J (2013). Identifying protein complexes from heterogeneous biological data. Proteins-Structure Function and Bioinformatics.

[17] Zhao JM, Hu XH, He TT, Li P, Zhang M, Shen XJ (2014). An edge-based protein complex identification algorithm with gene co-expression data (PCIA-GeCo). IEEE Transactions on Nanobioscience.

[18] Zhou HF, Liu J, Li JH, Duan WC (2017). A density-based approach for detecting complexes in weighted PPI networks by semantic similarity. PLoS One.

[19] Yang X-S (2012). Flower pollination algorithm for global optimization.

[20] Wang R, Zhou Y, Qiao S, Huang K (2016). Flower pollination algorithm with bee pollinator for cluster analysis. Inf Process Lett.

[21] Lei X, Fang M, Wu F-X, Chen L (2018). Improved flower pollination algorithm for identifying essential proteins. BMC Syst Biol.

[22] Ou-Yang L, Dai DQ, Li XL, Wu M, Zhang XF, Yang P (2014). Detecting temporal protein complexes from dynamic protein-protein interaction networks. BMC Bioinformatics.

[23] Zhang YJ, Lin HF, Yang ZH, Wang J, Liu YW, Sang ST (2016). A method for predicting protein complex in dynamic PPI networks. BMC Bioinformatics..

[24] Li M, Meng X, Zheng R, Wu FX, Li Y, Pan Y, Wang J. Identification of protein complexes by using a spatial and temporal active protein interaction network. IEEE/ACM Transactions on Computational Biology and Bioinformatics. 2017:1–1. 10.1109/TCBB.2017.2749571 28885159

[25] Wang JX, Peng XQ, Li M, Pan Y (2013). Construction and application of dynamic protein interaction network based on time course gene expression data. Proteomics.

[26] Zhao B, Wang J, Li M, Li X, Li Y, Wu FX, Pan Y (2016). A new method for predicting protein functions from dynamic weighted interactome networks. IEEE Trans Nanobioscience.

[27] Xenarios I, Salwinski L, Duan XJ, Higney P, Kim SM, Eisenberg DDIP (2002). The database of interacting proteins: a research tool for studying cellular networks of protein interactions. Nucleic Acids Res.

[28] Guldener U, Munsterkotter M, Oesterheld M, Pagel P, Ruepp A, Mewes HW, Stumpflen V (2006). MPact: the MIPS protein interaction resource on yeast. Nucleic Acids Res.

[29] Krogan NJ, Cagney G, Yu H, Zhong G, Guo X, Ignatchenko A, Li J, Pu S, Datta N, Tikuisis AP (2006). Global landscape of protein complexes in the yeast Saccharomyces cerevisiae. Nature.

[30] Tu BP, Kudlicki A, Rowicka M, McKnight SL (2005). Logic of the yeast metabolic cycle: temporal compartmentalization of cellular processes. Science (New York, NY).

[31] Binder JX, Pletscher-Frankild S, Tsafou K, Stolte C, O'Donoghue SI, Schneider R, Jensen LJ (2014). COMPARTMENTS: unification and visualization of protein subcellular localization evidence. Database-the Journal of Biological Databases and Curation.

[32] Cherry JM, Adler C, Ball C, Chervitz SA, Dwight SS, Hester ET, Jia Y, Juvik G, Roe T, Schroeder M (1998). SGD: Saccharomyces genome database. Nucleic Acids Res.

[33] Mewes HW, Frishman D, Mayer KF, Munsterkotter M, Noubibou O, Pagel P, Rattei T, Oesterheld M, Ruepp A, Stumpflen V (2006). MIPS: analysis and annotation of proteins from whole genomes in 2005. Nucleic Acids Res.

[34] Zhang R, Ou HY, Zhang CT (2004). DEG: a database of essential genes. Nucleic Acids Res.

[35] Pu SY, Wong J, Turner B, Cho E, Wodak SJ (2009). Up-to-date catalogues of yeast protein complexes. Nucleic Acids Res.

[36] Altaf-Ul-Amin M, Shinbo Y, Mihara K, Kurokawa K, Kanaya S (2006). Development and implementation of an algorithm for detection of protein complexes in large interaction networks. BMC Bioinformatics..

